# Clinical Outcomes and Treatment Characteristics of Seizure Threshold-Titrated Electroconvulsive Therapy in Depressive Disorder: A 3.5-Year Retrospective Naturalistic Course-Level Study

**DOI:** 10.3390/jcm15155890

**Published:** 2026-07-28

**Authors:** Claudia Elena Anghel, Ciprian Bacila, Bogdan Ioan Vintila, Monica Cornea, Andreea Maria Grama, Bianca Macavei, Andrei Lomnasan, Iulian Roman-Filip, Corina Roman-Filip

**Affiliations:** 1 Faculty of Medicine, Lucian Blaga University of Sibiu, 550024 Sibiu, Romania; claudia.anghel@ulbsibiu.ro (C.E.A.); bogdan.vintila@ulbsibiu.ro (B.I.V.); corina.roman@ulbsibiu.ro (C.R.-F.); 2 “Dr. Gheorghe Preda” Clinical Psychiatry Hospital of Sibiu, 550082 Sibiu, Romania; moni.sipos@ymail.com (M.C.); andreeamaria.grama@ulbsibiu.ro (A.M.G.); macavei_bianca@yahoo.com (B.M.); andreilomnasan96@gmail.com (A.L.); 3 Neuroscience Scientific Research Collective, 550082 Sibiu, Romania; 4 County Clinical Emergency Hospital of Sibiu, 550245 Sibiu, Romania; 5 Doctoral School, “George Emil Palade” University of Medicine, Pharmacy, Science and Technology, 540142 Târgu Mureș, Romania; iulian_roman2009@yahoo.com

**Keywords:** electroconvulsive therapy, treatment-resistant depression, seizure threshold, patient characteristics, global functioning, antidepressant response

## Abstract

**Background/Objectives**: Electroconvulsive therapy (ECT) remains the most effective biological treatment for treatment-resistant depression. Although its efficacy has been consistently demonstrated, the influence of seizure characteristics and treatment parameters on clinical outcomes in routine practice remains incompletely understood. This study aimed to evaluate the effectiveness of seizure threshold-titrated ECT in patients with treatment-resistant depression and to describe treatment outcomes in relation to seizure expression and technical treatment variables. **Methods**: We conducted a retrospective naturalistic study at the Department of ECT, Dr. Gh. Preda Clinical Psychiatric Hospital, Sibiu, Romania. Consecutive patients treated with ECT for treatment-resistant depressive episodes between March 2022 and December 2025 were included. Clinical outcomes were assessed before and after treatment using the Montgomery–Åsberg Depression Rating Scale (MADRS), Beck Depression Inventory (BDI), Mini-Mental State Examination (MMSE), and Global Assessment of Functioning Scale (GAF). Information regarding electrode placement, seizure threshold, seizure duration, the number of ECT sessions, and concomitant psychotropic medication was collected from medical records. **Results**: Nineteen acute ECT courses were available for outcome analysis. Depressive symptom severity improved significantly following treatment, with median MADRS scores decreasing from 38 (IQR 36–41) to 20 (IQR 9.5–24) (*p* < 0.001). A clinical response was observed in 57.9% of courses, while 36.8% achieved remission. Similar improvements were noted in self-reported depressive symptoms and overall functioning, with median BDI scores decreasing from 38 to 16 and median GAF scores increasing from 55 to 65. MMSE scores remained stable throughout treatment. Considerable variability was observed in both motor and EEG seizure durations; however, no consistent relationship between seizure duration and antidepressant response was identified. Discussion: The observed outcomes are consistent with previous evidence supporting ECT as an effective intervention for severe depressive illness. Functional improvement accompanied symptom reduction, while no decline in global cognitive performance, as assessed by the MMSE, was observed. In this cohort, seizure duration alone did not appear to provide meaningful information regarding treatment response. **Conclusions**: Seizure threshold-titrated ECT was associated with substantial clinical improvement in patients with treatment-resistant depressive episodes, accompanied by improved functioning and stable global cognitive performance. These findings support the continued use of individualized ECT protocols in routine clinical practice and highlight the need for further research on neurophysiological predictors of treatment outcome.

## 1. Introduction

Major depressive disorder (MDD) is a complex and highly varied psychiatric condition that affects an estimated 332 million people worldwide, and it is one of the leading causes of disability globally [1]. Over the past 30 years, the incidence of depression has increased by nearly 50% [2]. In addition to significantly impacting quality of life and daily functioning, depression is also closely linked to an increased risk of suicide. According to the World Health Organization (WHO), approximately 727,000 deaths were attributed to suicide in 2021, making it the third leading cause of mortality among individuals aged 15 to 29 years [3].

Despite the significant burden associated with depressive disorders, access to mental health care remains inadequate, with only about one-third of affected individuals in high-income countries receiving appropriate treatment [4]. Treatment-resistant depression (TRD) is a serious form of major depressive disorder in which patients do not achieve remission after trying two or more adequate courses of first-line antidepressant treatments. Approximately 30% of individuals diagnosed with major depressive episodes will experience TRD [5].

Over the past few decades, several evidence-based treatment options have become available for patients with depressive disorders, including pharmacotherapy, psychotherapy, bright light therapy (BLT), and neuromodulation techniques such as transcranial magnetic stimulation (TMS) [6,7,8]. Pharmacotherapy and psychotherapy remain first-line interventions for most patients; however, a substantial proportion of patients continue to experience clinically significant depressive symptoms despite adequate therapeutic interventions, highlighting the need for alternative treatment strategies.

Since its introduction in the late 1930s, electroconvulsive therapy (ECT) has remained the longest-established neuromodulation intervention and was introduced decades before modern antidepressant agents became available [9,10]. Among currently available biological interventions, ECT continues to occupy a central role in the management of severe depressive episodes, particularly in patients with treatment-resistant depression, psychotic features, significant functional impairment, or an urgent need for rapid clinical improvement, which results in improving the quality of life [11,12,13]. As the first line of treatment, ECT is recommended for treating patients at a high risk of suicide [14]. Despite its well-established efficacy and safety, several clinical and technical factors may influence treatment outcomes, including electrode placement, seizure threshold, seizure characteristics, and concomitant pharmacological treatment [15,16,17].

For patients with treatment-resistant depression, neuromodulation approaches such as transcranial magnetic stimulation and electroconvulsive therapy have gained increasing clinical relevance. Although TMS is generally well tolerated and has demonstrated antidepressant efficacy [18,19,20], comparative studies and meta-analyses consistently report higher response and remission rates with ECT, particularly in severe, psychotic, or highly treatment-resistant depressive episodes [21,22,23]. Furthermore, ECT is characterized by a rapid onset of action, making it especially valuable in situations requiring urgent clinical improvement, including severe suicidality, psychotic depression, or catatonia [22,23,24]. Consequently, despite the development of newer therapeutic modalities, ECT continues to be regarded as the gold-standard treatment for severe treatment-resistant depression [23,24].

Despite its documented efficacy, the dissemination of ECT remains constrained by limited availability and persistent concerns related to possible cognitive adverse effects [25,26]. As a result, ECT is typically reserved for patients experiencing severe symptoms, such as psychotic or catatonic features, or for those who do not respond adequately to pharmacological interventions [27,28]. Despite the considerable evidence supporting the significant therapeutic effects of ECT for various mental disorders, experts agree that it is underutilized [29,30,31,32,33,34,35,36]. In Romania, access to ECT remains particularly limited, with only a small number of centers currently providing modern ECT services. This underuse is often attributed to ongoing stigma and a lack of awareness about modern ECT techniques and their risk–benefit ratio [31,37,38,39,40]. Although ECT is an invasive procedure, established standards for ECT and anesthesia are in place to ensure optimal safety during treatment [41,42,43]. In contemporary ECT practice, stimulus dosing is individualized to the patient’s seizure threshold, defined as the lowest electrical charge that produces an adequate generalized seizure [15,16,17]. Although an adequate generalized seizure is essential for the therapeutic efficacy of ECT, excessively prolonged seizures may represent a safety concern. Seizure activity lasting more than 120 s is generally considered prolonged and may increase the risk of progression to status epilepticus, requiring immediate clinical intervention [24,44,45,46]. Consequently, seizure duration remains an important parameter for monitoring and optimizing ECT treatment [24,44,45]. Both the therapeutic effectiveness of ECT and the risk of cognitive side effects are influenced by the extent to which the applied stimulus exceeds this threshold [47,48,49]. Therefore, personalized titration of the seizure threshold has become a fundamental aspect of contemporary ECT dosing strategies [15,16,17,47,48,49].

Evidence accumulated over the years indicates that seizure threshold exhibits considerable interindividual variability and may progressively increase throughout an ECT course. Clinical factors, such as older age and electrode placement on both sides of the brain, are consistently associated with higher initial seizure thresholds and dynamic changes over time [50,51].

In addition to demographic and technical factors, concomitant psychotropic medication may significantly influence seizure threshold and seizure expression. Anticonvulsants and mood stabilizers are generally associated with an increased seizure threshold, while the effects of benzodiazepines and other psychotropic agents remain less consistent across studies [52]. Large naturalistic analyses have shown that older age and the use of anticonvulsant medication are independently associated with higher initial seizure thresholds and a greater likelihood of threshold increases during the early phases of bilateral ECT [53]. These findings highlight the importance of considering medication status when interpreting ECT dosing and clinical outcomes.

Electrode placement also plays a crucial role in both seizure characteristics and clinical efficacy. Bilateral ECT is generally associated with higher seizure thresholds and potentially greater antidepressant efficacy, though at the cost of an increased risk of cognitive side effects. In contrast, unilateral ECT may offer a more favorable cognitive profile [54,55,56]. How electrode configuration, seizure expression, and concomitant pharmacotherapy jointly affect treatment response remains poorly understood, particularly outside controlled research settings.

Despite the growing body of research on individual determinants of ECT outcomes, relatively few naturalistic studies have simultaneously integrated multidimensional clinical assessments, seizure threshold titration, electrode placement, seizure parameters, and concomitant pharmacotherapy. Considering these factors together may provide a more complete picture of ECT effectiveness and help refine individualized treatment strategies [48,54,57].

The aim of the present retrospective naturalistic study was to describe the clinical outcomes of seizure threshold-titrated electroconvulsive therapy in patients admitted for treatment-resistant depression and to characterize the treatment course by documenting technical parameters, seizure expression, electrode placement, concomitant medication, and patient characteristics, which were used to contextualize the observed clinical outcomes rather than to support causal or predictive inference. The primary objective was to examine treatment-related longitudinal changes in depressive symptom severity, as measured by the Montgomery–Åsberg Depression Rating Scale (MADRS) and the Beck Depression Inventory (BDI), alongside changes in cognitive functioning and global functioning, as assessed by the Mini-Mental State Examination (MMSE) and the Global Assessment of Functioning Scale (GAF). Secondary objectives included determining treatment response and remission rates, as well as contextualizing clinical outcomes in relation to technical treatment parameters, seizure expression, electrode placement, concomitant psychotropic medication, and selected patient characteristics. Depressive symptom severity constituted the primary outcome measure and was hypothesized to decrease significantly following ECT relative to baseline. Global functioning was similarly expected to improve post-treatment, whereas cognitive functioning was hypothesized to remain relatively stable across the treatment course. Additionally, seizure parameters and medication status were expected to provide clinically relevant information on treatment adequacy and therapeutic response.

## 2. Materials and Methods

### 2.1. Study Design and Setting

This study was designed as a retrospective, naturalistic observational study conducted at the Department of ECT at the Dr. Gh. Preda Clinical Psychiatric Hospital in Sibiu, a department within the Scientific Research Collective in Neurosciences. The study included all consecutive patients who underwent electroconvulsive therapy for a treatment-resistant depression episode between March 2022–December 2025, covering a three and a half year period. The naturalistic design reflects routine clinical practice, with treatment decisions—including electrode placement, dosing, and concomitant pharmacotherapy—made according to standard clinical indications rather than an experimental protocol.

Participants were eligible for inclusion if they were aged 18 years or older and had a diagnosis of a treatment-resistant depressive episode associated with depressive disorder, bipolar disorder, or schizoaffective disorder, with or without psychotic features and/or suicidal thoughts or attempts, according to the International Classification of Diseases, 10th Revision (ICD-10) criteria. All patients had completed a full course of electroconvulsive therapy (ECT), and the initial seizure threshold (IST) had been determined through a standardized dose titration procedure during the first ECT session. Complete pre- and post-treatment clinical assessments were required for inclusion, including the Montgomery–Åsberg Depression Rating Scale (MADRS), Beck Depression Inventory (BDI), Mini-Mental State Examination (MMSE), and Global Assessment of Functioning Scale (GAF). In addition, documentation of motor and electroencephalographic (EEG) seizure durations for each ECT session, as well as detailed information regarding concomitant psychotropic medication during the ECT course, had to be available.

Patients were excluded if they had incomplete clinical or ECT-related data, discontinued the ECT course prematurely without a post-treatment assessment, or developed neurological or other severe medical conditions that could significantly affect somatic or cognitive evaluation. Patients who withdrew informed consent during ECT treatment were also excluded, as were patients who were subsequently hospitalized with formal involuntary admission procedures.

The initial eligible sample consisted of 23 ECT courses contributed by 15 unique patients who met the eligibility criteria, as some patients were admitted on more than one occasion for clinically distinct treatment-resistant depression episodes. Of these 23 courses, three continuation/maintenance ECT courses were excluded from the acute-course analyses. One additional acute course was discontinued due to ECT-associated cardiovascular complications associated with an underlying cardiac condition, after which the patient continued treatment with 20 sessions of transcranial magnetic stimulation (TMS); this course was excluded from the primary outcome analysis. The primary analytic sample therefore consisted of 19 acute ECT courses.

### 2.2. Ethical Considerations

The study was conducted in accordance with the Declaration of Helsinki and was approved by the Institutional Review Board/Ethics Committee of the Dr. Gh. Preda Clinical Psychiatric Hospital. Given the retrospective nature of the study and the use of anonymized clinical data, informed consent for the use of clinical information for research purposes was obtained from patients at the time of admission, in accordance with institutional regulations.

### 2.3. ECT Procedure

#### 2.3.1. Equipment

ECT was administered using a brief-pulse, constant-current device in accordance with established clinical guidelines. Treatments were generally delivered two to three times per week, depending on clinical indications and patient tolerability.

#### 2.3.2. Anesthesia and Muscle Relaxation

All ECT sessions were performed under general anesthesia. The anesthetic agent used was Propofol, administered intravenously according to an anesthesiologist’s assessment. Succinylcholine (0.5–1.0 mg/kg) was used as a muscle relaxant to minimize motor manifestations of the seizure. Standard physiological monitoring included electrocardiography, non-invasive blood pressure measurement, and pulse oximetry.

#### 2.3.3. Electrode Placement

Electrode placement was determined by the treating psychiatrist based on clinical considerations. Patients received either right unilateral (RUL) ECT or bilateral (BL) ECT, including bitemporal or bifrontotemporal placement. The electrode placement type was recorded for subsequent comparative analyses.

#### 2.3.4. Determination of the Initial Seizure Threshold

The initial seizure threshold (IST) was determined during the first ECT session using a standardized dose titration protocol. Incremental electrical stimuli were administered until an adequate generalized seizure was elicited. The stimulus level required to induce this seizure was recorded as the patient’s seizure threshold. Subsequent treatments were delivered at suprathreshold stimulus intensities, in accordance with clinical guidelines and individualized patient response.

#### 2.3.5. Number of ECT Sessions

The total number of ECT sessions was individualized based on clinical response, tolerability, and the treating psychiatrist’s judgment. Treatment was continued until remission, significant clinical improvement, or no further clinically meaningful progress was observed.

### 2.4. Seizure Monitoring

#### 2.4.1. Motor Seizure Duration

Motor seizure activity was assessed using the movement sensor and visually monitored by the clinical team. The duration of motor seizures was recorded in seconds for each ECT session.

#### 2.4.2. EEG Seizure Duration

Electroencephalographic (EEG) monitoring was performed during each ECT session using the device’s integrated EEG system. EEG seizure duration was defined as the time from the onset to the termination of ictal EEG activity. For each patient, the average motor and EEG seizure durations across the treatment course were calculated. These analyses were descriptive and exploratory in nature and were performed to characterize seizure-duration trajectories rather than to establish predictive or causal associations with clinical outcomes.

### 2.5. Clinical and Cognitive Assessments

Patients were evaluated at two time points—baseline, before the initiation of the ECT course, and endpoint, generally 48–72 h after the final ECT session—thereby avoiding assessment during the immediate postictal period.

#### 2.5.1. Depressive Symptom Severity

The Montgomery–Åsberg Depression Rating Scale (MADRS) was used as a clinician-rated measure of depressive symptom severity, whereas the Beck Depression Inventory (BDI) was used as a self-reported measure of depressive symptoms. Together, these complementary instruments provided clinician-rated and patient-reported assessments of symptom severity and treatment-related change. These instruments were selected to assess depressive symptom severity and treatment-related change in patients with depressive disorders.

#### 2.5.2. Cognitive Functioning

The Mini-Mental State Examination (MMSE) was the standardized cognitive screening instrument routinely used in the clinical setting during the study period and, therefore, represented the only cognitive measure available for retrospective analysis.

#### 2.5.3. Global Functioning

The Global Assessment of Functioning Scale (GAF) was used to assess overall psychological, social, and occupational functioning.

### 2.6. Concomitant Psychotropic Medication

Information regarding concomitant psychotropic medication was extracted from medical records at the time of ECT initiation and reviewed throughout the ECT treatment course using medication administration records. Medications were categorized as antidepressants, antipsychotics, mood stabilizers/anticonvulsants, and benzodiazepines. The administered psychotropic regimen, including medication type and dosage, was compared with the regimen documented at baseline to identify any changes during the ECT course. Overall, the core psychotropic treatment regimen remained stable throughout the ECT course. These data were collected to characterize concomitant pharmacological treatment and to contextualize the observed clinical outcomes.

### 2.7. Outcome Measures

#### 2.7.1. Primary Outcome

The primary outcome measure was the change in depressive symptom severity, defined as the difference between baseline and endpoint MADRS scores (ΔMADRS).

#### 2.7.2. Secondary Outcomes

Secondary outcomes included changes in BDI, MMSE, and GAF scores, as well as treatment response, defined as a ≥50% reduction in the MADRS score from baseline; partial response, defined as a 30–49% reduction in the MADRS score from baseline; and remission, defined as a post-treatment MADRS score ≤10, associations between electrode placement and clinical outcomes, associations between the initial seizure threshold and treatment outcomes, associations between motor and EEG seizure durations and clinical improvement, and the influence of concomitant psychotropic medication on seizure threshold and treatment outcomes.

### 2.8. Variables of Interest

#### 2.8.1. Demographic and Clinical Variables

Demographic and clinical variables included age, sex, psychiatric diagnosis (major depressive disorder or bipolar depression), the duration of illness when available, previous ECT when available, and the presence of psychotic features when available.

#### 2.8.2. ECT-Related Variables

ECT-related variables included electrode placement (unilateral versus bilateral), initial seizure threshold, total number of ECT sessions, average motor seizure duration, and average EEG seizure duration.

## 3. Results

Analyses were performed at the level of the ECT course/admission. During the study period, 23 ECT courses corresponding to 15 unique patients were identified as eligible for review. Some patients contributed more than one course because they were admitted on separate occasions, more than one year apart, for clinically distinct treatment-resistant depressive episodes. These repeated admissions were therefore treated as distinct course-level treatment episodes, each with its own baseline assessment. ECT parameters, seizure duration data, and post-treatment outcome assessment were excluded from the acute-course analyses. There were three continuation/maintenance courses. One additional acute course was interrupted after 3 sessions due to detected cardiac arrhythmias, and this admission was excluded from the primary outcome analysis. The remaining acute admissions were assessed for the availability of evaluable longitudinal clinical outcome data and comprised the primary analytic sample. The primary analytic sample therefore comprised 19 acute ECT courses. R programming language (version 4.5.1; R Core Team, 2025) was used for data processing and analyses.

Within the included sample, the cohort was predominantly female, from urban areas, married, employed, and affected by recurrent depressive disorder. The baseline demographic and clinical characteristics of the 19 acute courses are shown in Table 1.

**Figure 1:**
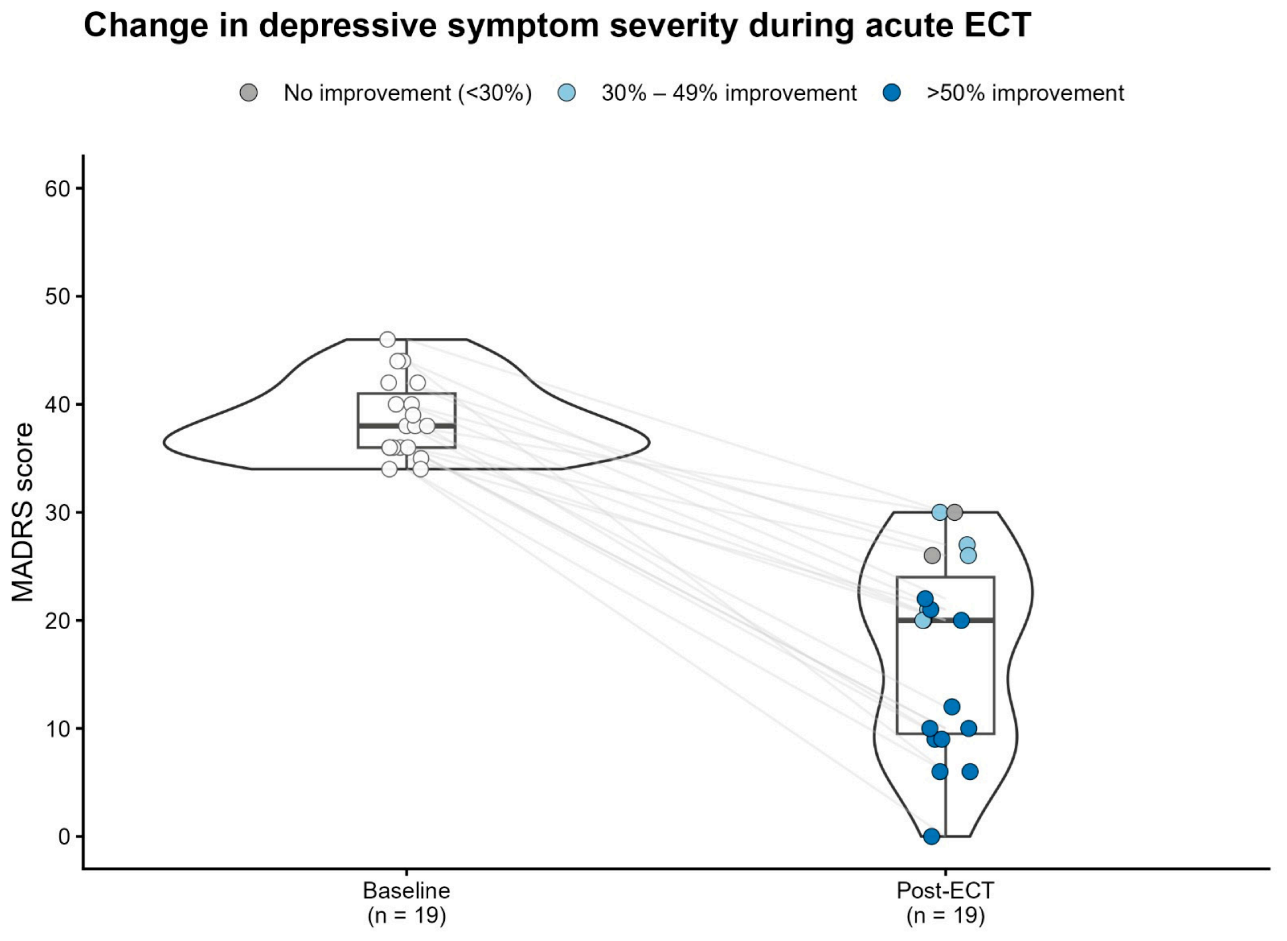
Change in depressive symptom severity. Violin–box plots show the distribution of MADRS scores at baseline and post-ECT for the 19 acute ECT courses. Thin lines connect paired observations within individual courses. Post-treatment points are colored according to the degree of clinical improvement.

Depressive symptom severity was lower post treatment than at baseline (Figure 1). The median MADRS score decreased from 38 (IQR, 36–41) to 20 (IQR, 9.5–24) after ECT. This reduction was significant on two-sided Wilcoxon signed-rank testing (V = 190; *p* < 0.001). The Hodges–Lehmann estimate for the paired reduction was 21 points (95% CI, 17–26), indicating a substantial median decrease in clinician-rated depressive symptom severity during the acute ECT course. At the individual-course level, 11 of 19 courses (57.9%) met criteria for a therapeutic response, defined as a greater than 50% reduction in MADRS score from baseline; 6 courses (31.6%) met criteria for partial response (30–49% reduction); and 2 courses (10.5%) showed no clinically meaningful improvement (<30% reduction). Using a post-treatment MADRS threshold of 10 or less, 7 out of 19 courses (36.8%) met the criteria for remission. 

**Figure 2:**
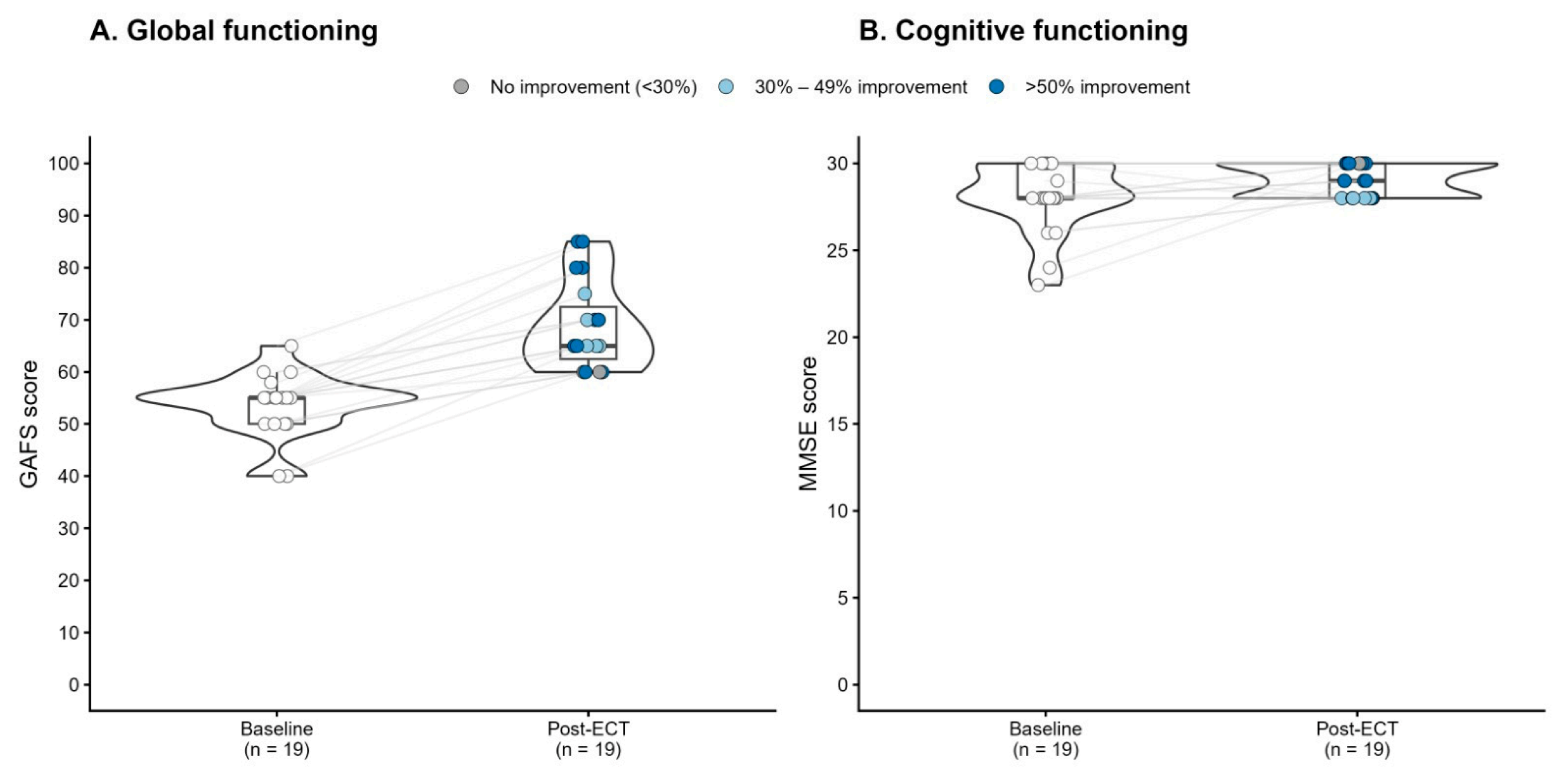
Changes in global functioning (**A**) and cognitive functioning (**B**). Violin–box plots show the distribution of GAF and MMSE scores at baseline and post-ECT for the 19 acute ECT courses. Thin lines connect paired observations within individual courses. Post-treatment points are colored according to the degree of clinical improvement.

Changes in secondary clinical measures, while expected, were interpreted descriptively. The modest sample size and observational design limited formal inference for these secondary outcomes. The median BDI score decreased from 38 (IQR, 33–39) to 16 (IQR, 9–25), the median GAF score increased from 55 (IQR, 50–55) to 65 (IQR, 62.5–72.5), and the median MMSE scores remained stable from baseline to post-treatment assessment (28 vs. 29) (Figure 2), suggesting no detectable decline in global cognitive performance as measured by the MMSE. However, given the limited sensitivity of the MMSE to specific cognitive domains, these findings do not exclude potential ECT-related changes in autobiographical memory, verbal memory, processing speed, or executive functioning.

ECT was delivered predominantly with right unilateral electrode placement, whereas bilateral and mixed placement accounted for a smaller proportion of cases (Table 2). Right unilateral electrode placement was the predominant treatment approach in this cohort, reflecting routine clinical practice at our institution. This approach was generally preferred because it is associated with a lower risk of cognitive adverse effects while maintaining established antidepressant efficacy when administered at adequate suprathreshold stimulus doses. Bilateral electrode placement was used when considered clinically appropriate by the treating psychiatrist. Initial stimulation settings were correspondingly clustered around brief-pulse, 20 Hz protocols, with broader pulse widths and longer stimulus durations observed mainly in the bilateral and mixed courses. Baseline pharmacotherapy reflected substantial clinical complexity, with most courses initiated in the context of a combination of psychotropic treatment, and nearly half involving medications from 3 psychotropic classes. Concurrent antidepressant and antipsychotic treatment were present in more than two-thirds of courses. Concomitant treatment was documented as unchanged during all included ECT courses, indicating that the observed clinical outcomes occurred against a stable background of psychopharmacologic treatment (Table 2).

**Figure 3:**
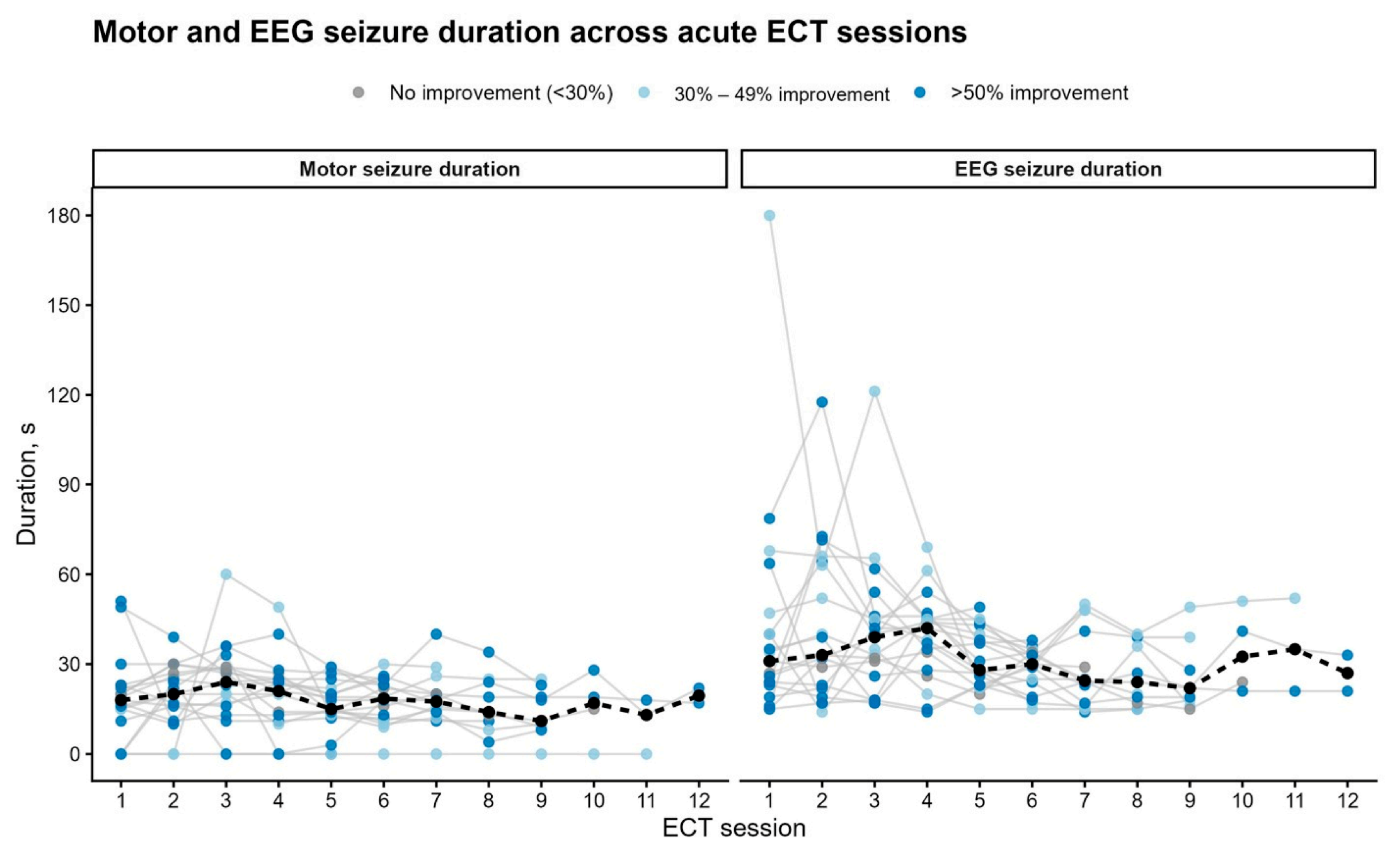
Motor and EEG seizure duration across acute ECT sessions. Thin gray lines connect individual acute ECT courses; points are colored according to the MADRS improvement category. The dashed black line and dots represent the overall median at each session. Because the number of courses without clinically meaningful improvement was small, visual comparisons by improvement category are descriptive only.

The session-level expression of induced seizures is shown in Figure 3. Across acute ECT courses, both motor and EEG seizure durations showed substantial within-course and between-course variability over successive treatment sessions. Most observations clustered in the lower- to mid-range of motor seizure duration, whereas EEG seizure durations were generally longer and exhibited greater variability, particularly in earlier sessions. Figure 3 also depicts color-coded courses based on MADRS-derived improvement categories. Seizure duration trajectories did not show clear visual separation by clinical outcome in this modest sample. However, these observations are descriptive and hypothesis-generating.

Overall, these findings indicate that acute seizure threshold-titrated ECT in this naturalistic cohort was associated with a marked reduction in depressive symptom severity and improved global functioning, while cognitive screening scores remained broadly stable. The technical and session-level seizure data demonstrated substantial heterogeneity across courses, but in this sample, they did not suggest (descriptively) a relation between seizure duration trajectories and antidepressant outcomes.

## 4. Discussion

This 3.5-year retrospective study evaluated the clinical outcomes of seizure threshold-titrated electroconvulsive therapy (ECT) in patients with depressive disorders, with particular emphasis on technical parameters, seizure duration, and patient characteristics.

Several clinically relevant findings emerged. First, acute ECT was associated with a marked reduction in depressive symptom severity [58], with median MADRS scores decreasing from 38 (IQR 36–41) at baseline to 20 (IQR 9.5–24) after treatment. The median paired reduction was 21 points, and 57.9% of treatment courses met criteria for clinical response, while 36.8% achieved remission. Second, improvement was also observed in self-reported depressive symptoms and global functioning, with median BDI scores decreasing from 38 to 16 and median GAF scores increasing from 55 to 65. The observed improvement in global functioning further supports the clinical relevance of the antidepressant response. Symptom reduction alone does not necessarily imply functional recovery; therefore, the concurrent improvement in both BDI and GAF scores suggests that ECT was associated not only with reduced depressive symptom severity but also with improved overall functioning and daily life performance [59,60,61]. Third, cognitive screening performance remained stable, with median MMSE scores changing only from 28 to 29. Although no decline in global cognitive performance, as assessed by the MMSE, was detected in our cohort, this finding should be interpreted with caution. Concerns about cognitive adverse effects remain among the main factors limiting the acceptance and use of ECT, and the stability of MMSE scores should be interpreted as indicating no detectable decline in global cognitive performance rather than the absence of ECT-related cognitive effects [24,62].

Finally, despite substantial variability in motor and EEG seizure durations across sessions, seizure duration trajectories did not visually distinguish responders from non-responders. Although these observations are descriptive, they are consistent with accumulating evidence suggesting that seizure duration alone may be an insufficient marker of ECT treatment adequacy or antidepressant outcome [24,63,64].

The antidepressant improvement observed in this cohort is consistent with the established role of ECT as one of the most effective interventions for severe, psychotic, or treatment-resistant depressive episodes [64,65,66,67,68]. In a recent national Scottish cohort of 2074 patients treated with ECT for moderate-to-severe depressive illness, Semple et al. reported response and remission rates were 73% and 51%, respectively [64]. Although the response and remission rates in our cohort were lower, they remain clinically meaningful considering the naturalistic design, small sample size, recurrent depressive disorder in 89.5% of courses, somatic comorbidity in 78.9%, and substantial baseline psychotropic polypharmacy. These differences may reflect greater clinical complexity, treatment resistance, comorbidity burden, and variability in outcome definitions across studies.

The observed improvement in functioning further supports the clinical relevance of the antidepressant response. Median GAF scores increased from 55 to 65, indicating that symptom reduction was accompanied by functional gains. Similarly, the decrease in BDI scores from 38 to 16 suggests that clinician-rated improvement was paralleled by improvement in patient-reported depressive symptoms. This convergence across clinician-rated, self-rated, and functional measures strengthens the interpretation that ECT produced meaningful clinical benefit rather than only statistical improvement.

A key objective of this study was to explore the relationship between seizure duration and antidepressant outcome. Historically, seizure duration has been used as a marker of treatment adequacy, but contemporary evidence suggests that duration alone is an imperfect indicator of therapeutic efficacy. In our cohort, motor and EEG seizure durations showed substantial within-course and between-course variability, but no clear visual separation was observed between response categories. This finding should be interpreted with caution, given the limited sample size and the descriptive nature of the analysis. Importantly, recent large-registry data indicate that seizure duration may still carry prognostic information. Gillving et al., in a cohort of 6998 patients with major depressive disorder, found that EEG seizure duration was associated with remission, with the highest remission rates observed for EEG seizures lasting 60–69 s [63]. Therefore, our findings do not refute the potential relevance of seizure duration, but rather suggest that in a small real-world cohort, seizure duration trajectories alone were insufficient to distinguish clinical outcomes.

The discrepancy between our descriptive observations and findings from larger registries may be explained by differences in statistical power, treatment protocols, concomitant medications, electrode placement, and sample composition. It is also possible that seizure duration is best understood as one component of treatment adequacy rather than as a standalone predictor. Contemporary models increasingly emphasize seizure quality, postictal suppression, EEG morphology, autonomic response, and broader neurophysiological markers as potentially more informative than duration alone [63,69]. Future studies with larger samples and detailed EEG-derived seizure quality indices may better clarify which seizure features are most relevant for antidepressant response.

The technical parameters used in this cohort reflect modern seizure threshold–based ECT practice. Most treatment courses were delivered using right unilateral electrode placement, and most were initiated with an ultrabrief pulse width of 0.3 ms. This strategy is generally intended to individualize the dose relative to the seizure threshold while reducing cognitive burden. Prior literature supports the importance of optimizing electrode placement, pulse width, and stimulus dose to balance efficacy and tolerability [15,70,71,72,73,74]. Right unilateral ultrabrief-pulse ECT has been associated with favorable cognitive outcomes, although some trials and meta-analyses suggest that ultrabrief stimulation may require more treatments or may be less rapidly effective than brief-pulse approaches in some populations [71,72,73,74]. In the present study, the predominance of right unilateral ultrabrief stimulation likely limited the ability to detect differences in outcomes between technical configurations.

The preservation of cognitive screening performance is another important finding. Median MMSE scores remained stable from baseline to post treatment, increasing from 28 to 29. Although the MMSE is not sensitive to subtle changes in autobiographical memory, verbal fluency, processing speed, or executive functioning, the absence of measurable deterioration is reassuring. This finding is consistent with the literature suggesting that modern ECT techniques, particularly right unilateral and ultrabrief-pulse approaches, may reduce cognitive adverse effects compared with traditional bilateral brief-pulse protocols [70,71,72,73,74,75,76]. Nevertheless, cognitive safety should not be overstated. Systematic reviews indicate that ECT may be associated with short-term cognitive impairment and autobiographical memory loss in some patients, and more detailed neuropsychological assessment is recommended when cognitive outcomes are a primary endpoint [75,76,77]. Therefore, future prospective studies should include instruments more sensitive than MMSE, particularly measures of autobiographical memory and executive function.

The naturalistic character of this cohort is both a limitation and a strength. Most patients were treated in the context of substantial clinical complexity, including psychiatric comorbidity, somatic comorbidity, and concomitant antidepressant, antipsychotic, and mood stabilizer. Importantly, pharmacological regimens were recorded as unchanged across the included ECT courses, which reduces the likelihood that the observed improvement was primarily attributable to medication modification. This real-world context enhances external validity and reflects the type of patients commonly encountered in psychiatric practice, although it also introduces heterogeneity that limits causal inference.

Several limitations should be acknowledged. First, the retrospective design limits causal interpretation and may introduce selection and information bias. Second, the small sample size restricted statistical power and precluded robust multivariable modeling of predictors. The unit of analysis was the ECT course rather than the individual patient. Although repeated admissions represented clinically distinct treatment-resistant depressive episodes requiring separate ECT courses, some patients contributed more than one treatment course. Therefore, the findings should be interpreted as descriptive and hypothesis-generating.

The findings should also be interpreted in the context of the limited availability of ECT services in Romania. Despite being considered the gold-standard treatment for severe treatment-resistant depression, ECT remains underutilized worldwide and is currently available in only a small number of Romanian psychiatric centers. During the study period, modern ECT was routinely performed in only two active centers nationwide, substantially limiting the number of eligible patients. Therefore, the present cohort reflects the practical realities of ECT delivery in Romania and provides valuable real-world data from a setting in which access to this treatment remains restricted. Third, the study analyzed ECT courses rather than unique patients. Although a small number of patients contributed more than one course, these corresponded to distinct depressive episodes occurring during separate hospital admissions. Consequently, the findings should be interpreted as reflecting episode-specific treatment outcomes.

Fourth, the absence of a control group prevents definitive attribution of improvement exclusively to ECT. Fifth, seizure-quality measures beyond motor activity and EEG duration were unavailable. Finally, cognitive outcomes were assessed solely using the MMSE, which is primarily a global cognitive screening instrument and may not be sufficiently sensitive to detect ECT-related changes in specific cognitive domains, including autobiographical and verbal memory, executive functioning, and processing speed. Although MMSE scores remained stable, this finding should be interpreted as indicating no detectable decline in global cognitive performance as measured by the MMSE and should not be considered evidence of the absence of ECT-related cognitive adverse effects. The lack of more sensitive cognitive measures, such as the Montreal Cognitive Assessment (MoCA) or a comprehensive neuropsychological battery, represents an important limitation of the present study.

Despite these limitations, this study provides clinically relevant real-world evidence regarding seizure threshold-titrated ECT in depressive disorders. The findings support the effectiveness and apparent cognitive tolerability of contemporary individualized ECT practice delivered predominantly using right unilateral ultrabrief stimulation. While seizure duration varied substantially across courses, it did not appear to function as a standalone clinical marker of antidepressant response in this small cohort.

Taken together, these limitations highlight the need for greater methodological standardization in ECT research. Comparable concerns have been identified in evaluations of other EEG-based interventions, in which protocol heterogeneity, limited assessment of statistical power and bias, and insufficient data transparency were found to undermine reproducibility [78]. In parallel, large-scale collaborations have demonstrated the feasibility of harmonized data collection and reusable anonymized datasets [79]. Accordingly, future prospective studies should include larger sample sizes, standardized seizure-quality metrics, transparent analyses, detailed cognitive testing, and longer follow-up periods to clarify how technical parameters and patient-level characteristics influence acute response, remission, and relapse after ECT [58,80].

## 5. Conclusions

In this naturalistic retrospective cohort, acute seizure threshold-titrated ECT was associated with significant reductions in depressive symptom severity in patients with treatment-resistant depressive episodes. Most patients achieved clinically meaningful improvement, while a substantial proportion met the criteria for remission. Improvements in depressive symptoms were accompanied by better global functioning, whereas global cognitive functioning remained largely stable throughout treatment. No clinically meaningful decline in global cognitive functioning, as measured by the MMSE, was observed. However, subtle cognitive adverse effects cannot be excluded because more sensitive cognitive measures were not available. Seizure duration trajectories did not show clear visual separation by clinical outcome category in this modest sample; therefore, these observations should be interpreted descriptively and regarded as hypothesis-generating rather than confirmatory. Consistent with this observation, variability in motor and EEG seizure durations did not demonstrate a clear relationship with antidepressant response, suggesting that seizure duration alone may have limited value as an isolated indicator of clinical outcome. Overall, these findings provide a descriptive characterization of clinical outcomes and treatment course features associated with individualized seizure threshold-titrated ECT in routine clinical practice. Future prospective studies with larger samples, comprehensive neuropsychological assessments, and detailed measures of seizure quality are needed to better characterize the relationship between seizure characteristics, treatment response, and cognitive outcomes.

## Figures and Tables

**Table 1:** Baseline demographic and clinical characteristics.

Characteristic	N = 19
Age, y, Median (Q1, Q3)	41 (38, 47)
Sex, n (%)	
Female	15 (78.9%)
Male	4 (21.1%)
Residence, n (%)	
Urban	15 (78.9%)
Rural	4 (21.1%)
Marital status, n (%)	
Married	13 (68.4%)
Unmarried	6 (31.6%)
Occupational/social category, n (%)	
Employed	14 (73.7%)
Retired	4 (21.1%)
Student	1 (5.3%)
Primary diagnosis, n (%)	
Bipolar depression	1 (5.3%)
Recurrent depressive disorder	17 (89.5%)
Schizoaffective depression	1 (5.3%)
Any psychiatric comorbidity, n (%)	6 (31.6%)
Any somatic comorbidity, n (%)	15 (78.9%)
ECT sessions, No., n (%)	
6	5 (26.3%)
7	3 (15.8%)
8	1 (5.3%)
9	6 (31.6%)
10	1 (5.3%)
11	1 (5.3%)
12	2 (10.5%)
Repeat admission during study period, n (%)	8 (42.1%)

Values are median (IQR) for continuous variables and n (%) for categorical variables. Acute ECT admissions only; continuation/maintenance courses excluded.

**Table 2:** ECT course characteristics and concomitant psychotropic treatment.

Characteristic	N = 19
Electrode placement, n (%)	
Right unilateral	14 (73.7%)
Bilateral	3 (15.8%)
Mixed	2 (10.5%)
Stimulus number at treatment initiation, n (%)	
2	11 (57.9%)
3	7 (36.8%)
5	1 (5.3%)
Pulse width at treatment initiation, ms, n (%)	
0.3	15 (78.9%)
1	4 (21.1%)
Frequency at treatment initiation, Hz, n (%)	
20	17 (89.5%)
30	2 (10.5%)
Stimulus duration at treatment initiation, s, n (%)	
2	11 (57.9%)
3	2 (10.5%)
4	4 (21.1%)
8	2 (10.5%)
Antidepressants at baseline, No., n (%)	
1	7 (36.8%)
2	7 (36.8%)
3	4 (21.1%)
11	1 (5.3%)
Any antipsychotic at baseline, n (%)	13 (68.4%)
Antipsychotics at baseline, No., n (%)	
0	6 (31.6%)
1	7 (36.8%)
2	6 (31.6%)
Any mood stabilizer/anticonvulsant at baseline, n (%)	13 (68.4%)
Psychotropic classes at baseline, No., n (%)	
1	2 (10.5%)
2	8 (42.1%)
3	9 (47.4%)

Values are median (IQR) for continuous variables and n (%) for categorical variables. Stimulation parameters reflect the setting at treatment initiation. Mixed courses indicate switching from right unilateral to bilateral placement during the course. Continuation/maintenance courses and the interrupted cardiac course were excluded. Concomitant treatment was recorded as unchanged during ECT in all included courses.

## Data Availability

Anonymized data are available from the corresponding author upon reasonable request.
